# Effective Universal Coverage of Diabetes Mellitus Type 2 in Chile

**DOI:** 10.1590/1518-8345.1630.2871

**Published:** 2017-04-06

**Authors:** Sara Guerrero-Núñez, Sandra Valenzuela-Suazo, Patricia Cid-Henríquez

**Affiliations:** 1 Doctoral student, Facultad de Enfermería, Universidad de Concepción, Concepción, Bío Bío, Chile. Professor, Facultad Ciencias de la Salud, Universidad de Atacama, Copiapó, Chile.; 2 PhD, Full Professor, Facultad de Enfermería, Universidad de Concepción, Concepción, Bío Bío, Chile.

**Keywords:** Diabetes Mellitus Type 2, Universal Coverage, Nursing, Primary Health Care

## Abstract

**Objective:**

determine the prevalence of Effective Universal Coverage of Diabetes Mellitus Type 2 in Chile and its relation with the variables: Health Care Coverage of Diabetes Mellitus Type 2; Average of diabetics with metabolic control in 2011-2013; Mortality Rate for Diabetes Mellitus; and Percentage of nurses participating in the Cardiovascular Health Program.

**Method:**

cross-sectional descriptive study with ecological components that uses documentary sources of the Ministry of Health. It was established that there is correlation between the Universal Effective Coverage of Diabetes Mellitus Type 2 and the independent variables; it was applied the Pearson Coefficient, being significant at the 0.05 level.

**Results:**

in Chile Universal Health Care Coverage of Diabetes Mellitus Type 2 (HbA1c<7% estimated population) is less than 20%; this is related with Mortality Rate for Diabetes Mellitus and Percentage of nurses participating in the Cardiovascular Health Program, being significant at the 0.01 level.

**Conclusion:**

effective prevalence of Universal Health Coverage of Diabetes Mellitus Type 2 is low, even though some regions stand out in this research and in the metabolic control of patients who participate in health control program; its relation with percentage of nurses participating in the Cardiovascular Health Program represents a challenge and an opportunity for the health system.

## Introduction

Definition of Universal Health Coverage: "system capacity to respond to the health needs of a population, which includes providing infrastructure, human resources, health technologies (including medicines), and financing"^1^. Governments are responsible for deciding which health services are necessary, ensuring their universal availability, affordability, effectiveness and quality^2^. The member states of the World Health Organization (WHO) committed to reach this Coverage in 2005, are convinced that all people should have access to the health services they need, without the risk of economic loss or impoverishment^2^.

Chile addressed this Coverage with strategies such as the Explicit Health Guarantees Regime (EHGR), contained in the law EHGR N° 19966^3^, which standardizes the care of prioritized pathologies, guaranteeing access, quality, opportunity and financial support to the patients who suffer from those pathologies. Diabetes Mellitus type 2 (DM2) is one of the guaranteed pathologies, due to its morbidity and mortality rate, and among others, to the economic expenses associated with complications. In order to respond to the needs of population, it is essential that (*prestaciones en salud garantizadas*) guaranteed health benefits be timely and good quality, that is to say, they must allowed to move from Universal Coverage to Effective Coverage, since universal health coverage is not in itself a guarantee of efficacy and efficiency of care provided^4^, it is better understood as the coverage of health services and protection against financial risks and it is still far from the goal of universal coverage^4^. For this reason, it is necessary to develop researches that design indicators for the evaluation of policy progress of universal health coverage^4^. A good indicator is the Universal Effective Health Coverage^5^, since it guarantees all, in an equal way, the maximum achievable level of health outcomes from a package of high quality services that also avoids financial crises through the reduction of out-of-pocket costs^6^. Effective coverage is a part of a potential health gain, offered to the population through the health system^5^, it not only considers a particular intervention or service, but also determines that they are necessary to produce the desired effect on the patient's health^7^. 

The global prevalence of DM2 in 2013 was 8.3% in adults, and it was projected to be 9.9% by 2030^8^. Forty six percent of people with DM2 have not been diagnosed^9^, which results in a complex situation, considering that in 2012 Diabetes Mellitus was the fifteenth cause of premature death in both sexes^10^. According to the 2009-2010 National Health Survey (NHS), the prevalence of DM2 in Chile was 9.4% in 2009. The Basic Health Indicators (Chile 2013) report, issued by the Health Statistics and Information Department (HSID), indicated that in 2013, 33.8% of diabetics participated in the health control program. For this reason, the objective of national strategies was to increase the proportion of people with controlled diabetes. It was established as a goal to increase in 20% the effective coverage of diabetes mellitus type 2; the following figures were proposed: 29.8% in 2010, 31.8% in 2015 and 35.8% in 2020^11^. Nurses who participate in the Cardiovascular Health Program (CVHP), care for people with DM2 according to the clinical guide and the list of guaranteed benefits. These guidelines barely account for the role of nursing, which causes the wrong replacement of those professionals. Nonetheless, scientific evidence demonstrates the significant role that nursing plays in the metabolic control of DM2^12^ ^-^ ^15^. 

The incorporation of the Universal Health Coverage in the public policy and the low metabolic control of diabetic people, guided the objective of this article, which is to determine the prevalence of Effective Universal Coverage of Diabetes Mellitus Type 2 in Chile and its relation with the variables: Health Care Coverage of Diabetes Mellitus Type 2, Average of diabetics with metabolic control in 2011-2013, Mortality Rate for Diabetes Mellitus, and Percentage of nurses participating in the Cardiovascular Health Program.

## Method

It is a cross-sectional descriptive study with ecological components, systematizing national statistics and from different regions of the country, during the years 2011 to 2013. Because the official statistics for the years 2014 and 2015 have not been published (only preliminary documents), this period was not considered in the research. The following reports, obtained from the Ministry of Health, were used as documentary sources: Basic health indicators - Chile 2013; Controlled population (cardiovascular health program) by Region and by the Health Service, SNSS 2011, 2012 and 2013; and Controlled population (cardiovascular health program) according to compensation goals, by Region and Health Service, SNSS 2011, 2012 y 2013, belonging to HSID. The study did not require authorization from the ethics committee or informed consent, since the HSID reports were digitally available for public consultation. 

This study verified the prevalence of Effective Universal Coverage of Diabetes Mellitus Type 2 in Chile and its relation with four independent variables. To evaluate the variables, we worked with the universe of the national and regional population and with the universe of diabetic patients participating in the health control program (Cardiovascular Health Program).

- Dependent variable: Effective Universal Coverage of Diabetes Mellitus Type 2 (Percentage of patients in metabolic control [HbA1c <7%], according to the prevalence of Diabetes Mellitus type 2 at the national and regional level). 

- Independent variables: important variables were selected with periodic measurement in the Cardiovascular Health Program, they were: 1) Health Care Coverage of Diabetes Mellitus Type 2 (Percentage of patients participating in the health control program, according to the prevalence of Diabetes Mellitus type 2 at the national and regional level); 2) Average of diabetics with metabolic control in 2011-2013 (mean percent of type 2 diabetic patients metabolically controlled, during the years 2011-2013, that participate in the health control program); 3) Mortality Rate for Diabetes Mellitus, which was selected because it corresponds to a measurement of the last effect produced by the disease that is of national and international importance; and 4) Percentage of nurses participating in the Cardiovascular Health Program (Proportion of care provided by nurses in the Cardiovascular Health Program). The latter variable was incorporated because of researchers interest; this, was due to the academic training of the researchers and to the scientific evidence about the relevant participation of nursing professionals in the metabolic control Diabetes Mellitus Type 2. 

Statistical analysis was performed using the statistical software SPSS 19.0, applying the Pearson's Coefficient with significant value to the level of 0.05.

## Results 

The descriptive statistics presented in Table 1, shows that the dependent variable is the one with the lowest variability (SD = 3.40%). In this regard, Table 2 shows that the region of Antofagasta has the lowest Effective Universal Coverage of Diabetes Mellitus Type 2 and the region of Biobío has the highest. The same dependent variable has an average (17.80%) that differs from that indicated in Table 2, since the national values were extracted from the HSID reports and not according to the calculation of the regional average. This situation also occurs in Table 3, which contains national values extracted from the same statistics and not according to the regional average. 

**Table 1:** Descriptive statistics of the study variables. Chile, 2013

Variables	N	Minimum	Maximum	Mean	Standard Deviation (SD)
Effective Universal Coverage of Diabetes Mellitus Type 2	15	10.81	21.72	17.80	3.40
Health Care Coverage of Diabetes Mellitus Type 2	15	29.13	44.49	35.02	4.64
Average of diabetics with metabolic control in 2011-2013	15	37.55	49.17	43.04	3.59
Mortality Rate for Diabetes Mellitus	15	10.88	27.25	17.90	4.58
Percentage of nurses participating in the Cardiovascular Health Program	15	16.13	56.71	29.08	11.20

**Table 2:** Regional and national distribution of population, Estimated Prevalence of Diabetes Mellitus Type 2, Metabolically controlled in 2013 and Universal Effective Health Coverage de Diabetes Mellitus type 2. Chile, 2013

Region	Population *	Estimated prevalence of DM2 ^†^	Metabolically controlled 2013^‡^	Universal Effective Health Coverage Diabetes Mellitus type 2 (%)^§^
Arica and Parinacota	179615	16883,81	3636	21,54
Tarapacá	336121	31595,37	4881	15,45
Antofagasta	594555	55888,17	6043	10,81
Atacama	286624	26942,65	5187	19,25
Coquimbo	749374	70441,15	11189	15,88
Valparaíso	1814079	170523,42	32704	19,18
Metropolitan region of Santiago	7069645	664546,63	107354	16,15
Libertador B. O´Higgins	908553	85403,98	17076	19,99
Maule	1031622	96972,46	20581	21,22
Biobío	2074094	194964,83	42355	21,72
La Araucanía	994380	93471,72	17285	18,49
Los Ríos	382741	35977,65	7116	19,78
Los Lagos	867315	81527,61	12401	15,21
Aisén (General Carlos Ibáñez del Campo)	107915	10144,01	1210	11,93
Magallanes and Chilean Antarctic	160164	15055,41	3065	20,36
Country	17.556815	1650340,61	292083	17,69

* Extracted from Basic Health Indicators report - Chile 2013

† Prevalence obtained from Health Goals and Improvement of Primary Health Care for the year 2014 (9,4% according to the National Health Survey 2009-2010); DM2 (Diabetes Mellitus Type 2)

‡ Extracted from the report Population being controlled, cardiovascular health program, according to compensation goals, by Region and Health Service, SNSS 2013 (Diabetic people attending health control and metabolically controlled with HbA1c <7%)

§( Metabolically controlled in 2013 x 100)/ Estimated Prevalence of DM2

**Table 3:** Regional and national distribution of Health Care Coverage of Diabetes Mellitus Type 2, Average of diabetics with metabolic control in 2011-2013, Mortality Rate for Diabetes Mellitus and Percentage of Nurses Participating in the Cardiovascular Health Program. Chile, 2013

Region	Coverage DM2 (%)*	Average of diabetics with metabolic control in 2011-2013 (%)^†^	Mortality rate DM^‡^	Percentage of Nurses Participating CVHP(%)§
Arica and Parinacota	41.28	46.19	24.56	39.81
Tarapacá	36.50	47.30	10.88	21.80
Antofagasta	44.49	38.22	11.52	16.13
Atacama	38.94	49.17	27.25	21.60
Coquimbo	31.52	41.12	14.68	21.68
Valparaíso	41.98	44.39	19.52	25.01
Metropolitan region of Santiago	33.36	40.90	22.76	25.40
Libertador B. O´Higgins	31.41	47.20	18.50	17.94
Maule	32.52	41.74	19.89	56.71
Biobío	31.89	40.62	18.84	29.62
La Araucanía	31.30	42.52	15.94	37.17
Los Ríos	33.85	38.84	16.29	37.17
Los Lagos	31.48	37.55	18.43	29.20
Aisén (General Carlos Ibáñez del Campo)	29.13	45.80	13.22	17.09
Magallanes and Chilean Antarctic	35.72	44.07	16.34	39.95
Country	33.80	41.77	19.86	26.62

* Extracted from "Basic Health Indicators - Chile 2013"; DM2 (Diabetes Mellitus type 2)

† Obtained from " Population in control, Cardiovascular health program for Region and Health Service, SNSS 2011, 2012 y 2013" and " Population in control, Cardiovascular health program, according to compensation goals, for Region and Health Service, SNSS 2011, 2012 y 2013"

‡ Extracted from "Basic Health Indicators - Chile 2013" (Rate calculated for 17,556,815 inhabitants); DM (Diabetes Mellitus)

§ Obtained from "Controls according to health problem, by type of control, Region and Health Service, SNSS 2013"; CVHP (Cardiovascular Health Program).

Regarding the independent variables, the percentage of nurses participating in the Cardiovascular Health Program has the highest variability (SD=11.20%); the region of Maule stands out with the highest percentage and the region of Antofagasta with the lowest. The variable Health Care Coverage of Diabetes Mellitus Type 2 (SD=4.64%), in the Antofagasta region has the highest percentage and the Aisén region (General Carlos Ibáñez del Campo) the lowest. The variable Mortality Rate for DM (SD=4.58 per 100,000 population), in the region of Atacama has the highest mortality rate and the region of Tarapacá the lowest. Finally, the average number of diabetics participating in metabolic control in 2011-2013 presented the lowest variability (SD=3.59%); the Atacama region has the highest percentage and the region of Los Lagos the lowest. 

Table 4 shows statistical relationships between Effective Universal Coverage of Diabetes Mellitus Type 2 and independent variables. The relationship with Mortality Rate for Diabetes Mellitus is significant at the 0.05 level and with the Percentage of nurses participating in the Cardiovascular Health Program is significant at the level of 0.01.

**Table 4:** Correlation between Effective Universal Coverage of Diabetes Mellitus Type 2 and independent variables. Chile, 2013

Correlation between variables		V.D*	V.I 1^†^	V.I 2^‡^	V.I 3§	V.I 4^||^
V.D*						
	Pearson's correlation	1	-0.043	0.237	0.591	0.642
	Sig. (bilateral)		0.878	0.396	0.020	0.010
N	15	15	15	15	15

*V.D (Effective Universal Coverage of Diabetes Mellitus Type 2) †V.I 1 (Health Care Coverage of Diabetes Mellitus Type 2)

‡V.I 2 (Average of diabetics with metabolic control in 2011-2013) §V.I 3 (Mortality Rate for Diabetes Mellitus)

||V.I 4 (Percentage of nurses participating in the Cardiovascular Health Program)

## Discussion

DM2 is a disease that has a cardiovascular risk, which makes it in a public health problem at the national and international level^16^. In Chile as in other countries the prevention and control of cardiovascular diseases is a health priority. The national coverage of DM2 is 33.80% (Table 3), which corresponds to the low percentage of patients participating in the Cardiovascular Control Health Program.

The effective coverage of Diabetes Mellitus type 2 in people, aged 15 years or more, is an indicator that represents progress in Universal Health Coverage policy for this disease; this indicator has allowed the incorporation of a measurement according to its prevalence. This coverage incorporates people with controlled or compensated DM2 (HbA1c <7%) according to the prevalence of the disease. Achieving this type of coverage requires a supply of good quality health services, according to the needs of the people, which contributes to improve the health of the people receiving the intervention. For this reason, it is necessary for people to be aware of their needs and make use of the services^7^ ^,^ ^17^; therefore reducing personal expenses for health ^18^.

In this context it is possible to situate the Effective Universal Coverage of Diabetes Mellitus Type 2, since it aims to reach HbA1c <7% in the estimated national population with DM2. In this coverage is implicit the concept of quality^5^. The coverage must guarantee health benefits to a universe of patients who need such care or health services, and not only to those who are registered. In Chile, the coverage of noncommunicable diseases is lower than that of other diseases, especially when measuring effective coverage^19^; for this reason it is fundamental to work on coverage of pathologies such as DM2. The Average of diabetics patients with metabolic control in 2011-2013, at the national level is 41.77%, but when considering the prevalence of DM2, it is evident that less than 20% reach the Universal Effective Health Coverage (17.69%). This level of coverage is a reality that does not differ among the country regions. For this reason and for epidemiological, social and health policy in the country, this indicator presents a clear risk of remaining at the same level, if persist inadequate lifestyles and lack of comprehensive health strategies that could impact on the diagnosis and treatment of DM2; the lack of participation of patients in the health control is another factor that must be improved. Achieving the metabolic control in these people is a challenge, because not all people are aware of their health needs, or else they are the result of differences in the quality of care received^17^. For this reason, key aspects in the management of DM2 with Universal Effective Health Coverage are: population and health systems must know the health needs; use of services in a timely manner; develop a quality management system. This should guarantee the standardization of services and the integrality in the control of the DM2.

The two regions with the highest average of diabetics with metabolic control in 2011-2013 (Atacama, Tarapacá) stand out with a Health Care Coverage of Diabetes Mellitus Type 2 higher than the national average. Nonetheless, the Atacama region shows the highest Mortality Rate for Diabetes Mellitus at the national level (27.25 per 100,000 inhabitants), which indicates that it is not being effective, due to several factors that generate the increase of this indicator. 

The Mortality Rate for Diabetes Mellitus presents a statistically significant relationship with the Effective Universal Coverage of Diabetes Mellitus Type 2, which increases as Coverage increases; this could be attributed to several variables, since that disease is associated with cardiovascular risk factors, as well as social determinants, which may increase the risk of becoming ill or dying. Similar results were published in a prestigious medical journal; the article indicates that the metabolic control of patients with DM2 is not always associated with decrease of cardiovascular events or mortality^20^. For that reason, it is important that the control of these patients could move from a health management focused on glucose to a management focused on cardiovascular risk factors^21^.

The variable Percentage of nurses participating in the Cardiovascular Health Program presents the greatest variability, this shows participation according to the availability of professionals, and also to the priorities raised by the health teams in their annual programming. This variable is statistically related to Effective Universal Coverage of Diabetes Mellitus Type 2, which increases as the coverage increases. The participation of nursing in interventions to achieve health goals can contribute effectively to the achievement of regional and global goals; the Universal Health Coverage is not an exception^22^. For this reason, The limited participation of nursing at the national level in the CVHP (26.62%) is an obstacle for the effective coverage, since the scientific evidence has demonstrated the important role that nursing has in the metabolic control of DM2^12^ ^-^ ^15^. 

The Explicit Guarantees Regime and the list of specific benefits are mainly oriented towards pharmacological aspects and medical care, limiting the participation of nurses to consultation or control by a nurse, midwife or nutritionist, to confirm the diagnosis and/or perform the initial evaluation of the patient with DM2. As for the list of benefits in the treatment for the first and second years, appears again the consultation or control by nurse, midwife or nutritionist, this time incorporating group education by nurse, midwife or nutritionist. These limitations and the non-determination of actions and/or the non-existence of quality standards aimed at comprehensive care, in addition to the equivocal substitution of the role of the nurse by another professional, determines the need to make changes in those benefits, which would improve the Universal Effective Health Coverage.

Proper management by professional nurses would strengthen the health system, since it will contribute to meet expectations of population, improving their health status, and will also impact the policy of Universal Health Coverage. In this way, this management intervenes positively in health problems, such as low metabolic control of diabetic patients: good functioning of the health system generates effective responses to public health problems and contributes to effective economic management. Just as in Latin America the profound socioeconomic inequalities and those of health, in the 1990s, demanded health reforms that should strengthened the Universal Health Coverage^23^, the current epidemiological, economic, social and political scenario, among others, demands the implementation of Effective Universal Coverage of Diabetes Mellitus Type 2, by means of the strengthening the current health benefits. To do this, systems need to improve: the base of knowledge, the combination of skills and the availability and distribution of health workers^24^.

This study presents limitations such as the ecological fallacy, since the results concentrate a general situation by region and at the country level, where the relationships among variables do not necessarily occur at the individual level. However, it is necessary to recognize that the relationship between the Effective Universal Coverage of Diabetes Mellitus Type 2 and the Percentage of nurses participating in the Cardiovascular Health Program could also be applied, considering scientific evidence that confirms the important role of nursing in the metabolic control of this pathology^12^ ^-^ ^15^. In this regard, it would of interest to address the variable percentage of nurses participating in the Cardiovascular Health Program, not only to know participation in health control, but also in other interventions, especially those belonging to the list of benefits, namely Group education in the treatment of the first and second year. Unfortunately, the public statistics of the HSID do not incorporate this information, which prevents a comprehensive analysis.

Given the current scenario, it is essential that Chilean APS responds to the great challenge of improving the Effective Universal Coverage of Diabetes Mellitus Type 2, addressing this health problem in a comprehensive manner, which may require that the list of health benefits be expanded. At the same time, it is necessary to encourage the active participation of professionals such as nurses in solving these problems, knowing that the insufficiency of human resources becomes an obstacle to achieve the universal coverage^25^. Such insufficiency must be understood not only in terms of number of professionals, but also in its distribution. For all the mentioned reasons, this study contributes to critically analyze the policy implementation of universal health coverage on this pathology, and facing the results found, to enrich the health system with a new list of benefits that will help to achieve the maximum benefit in health and welfare for people with DM2. In that way, the results allow to consider the management of care as a fundamental pillar in the Effective Universal Coverage of Diabetes Mellitus Type 2 and encourage the performance of professional nurses beginning with health promotion till treatment of the disease. 

## Conclusion

Effective Universal Coverage of Diabetes Mellitus Type 2 - understood in this study as the effectiveness of public policy in Universal Health Coverage with regard to Diabetes Mellitus type 2 in Chile - continues to be a challenge of health at the national level, due to its low prevalence, even though some regions is in timely diagnosis of this pathology. In accordance with this, it is important to advance not only towards universal coverage, but also with regard to effectiveness, because the results show that coverage of this pathology, even the metabolic control of patients attending the CVHP, are not related to a better effective coverage.

On the other hand, Effective Universal Coverage of Diabetes Mellitus Type 2 is positively related to participation of nursing professionals in the CVHP. The latter assertion becomes a challenge for nursing; it implies the need of increasing its participation in the care of the vulnerable population. Besides that, it also constitutes a health opportunity, since it would allow reorienting the annual programming of health, giving priority to strategies that impel an approach more active and participatory of nursing in the CVHP. The researchers, consider important to carry out new studies that address variables such as effectiveness and participation of nursing; but also that these studies be used to develop public policies aimed at the population aiming to achieve maximum potential in health. It is also essential to continue studying this type of coverage with new variables that strengthen the understanding of the phenomenon.
